# Motor Unit Characteristics after Targeted Muscle Reinnervation

**DOI:** 10.1371/journal.pone.0149772

**Published:** 2016-02-22

**Authors:** Tamás Kapelner, Ning Jiang, Aleš Holobar, Ivan Vujaklija, Aidan D. Roche, Dario Farina, Oskar C. Aszmann

**Affiliations:** 1 Institute for Neurorehabilitation Systems, Bernstein Center for Computational Neuroscience, University Medical Center Göttingen, Georg-August University, Göttingen, Germany; 2 Department of Systems Design Engineering, University of Waterloo, Waterloo, Canada; 3 Faculty of Electrical Engineering and Computer Science, University of Maribor, Maribor, Slovenia; 4 CD Laboratory for Restoration of Extremity Function, Division of Plastic and Reconstructive Surgery, Medical University of Vienna, Vienna, Austria; Universite de Nantes, FRANCE

## Abstract

Targeted muscle reinnervation (TMR) is a surgical procedure used to redirect nerves originally controlling muscles of the amputated limb into remaining muscles above the amputation, to treat phantom limb pain and facilitate prosthetic control. While this procedure effectively establishes robust prosthetic control, there is little knowledge on the behavior and characteristics of the reinnervated motor units. In this study we compared the *m*. *pectoralis* of five TMR patients to nine able-bodied controls with respect to motor unit action potential (MUAP) characteristics. We recorded and decomposed high-density surface EMG signals into individual spike trains of motor unit action potentials. In the TMR patients the MUAP surface area normalized to the electrode grid surface (0.25 ± 0.17 and 0.81 ± 0.46, p < 0.001) and the MUAP duration (10.92 ± 3.89 ms and 14.03 ± 3.91 ms, p < 0.01) were smaller for the TMR group than for the controls. The mean MUAP amplitude (0.19 ± 0.11 mV and 0.14 ± 0.06 mV, p = 0.07) was not significantly different between the two groups. Finally, we observed that MUAP surface representation in TMR generally overlapped, and the surface occupied by motor units corresponding to only one motor task was on average smaller than 12% of the electrode surface. These results suggest that smaller MUAP surface areas in TMR patients do not necessarily facilitate prosthetic control due to a high degree of overlap between these areas, and a neural information—based control could lead to improved performance. Based on the results we also infer that the size of the motor units after reinnervation is influenced by the size of the innervating motor neuron.

## Introduction

Targeted Muscle Reinnervation (TMR) has become an increasingly accepted method in prosthetic rehabilitation, particularly for high level amputations. TMR consists of transferring nerves previously innervating the amputated limb to muscles within the stump area. The transferred nerves then reinnervate the targeted muscles, enabling them to serve as biological amplifiers of the neural control signals. The procedure allows for the use of multiple degrees of freedom prosthetic devices for patients who would otherwise only be capable of using prostheses with very limited capabilities [1].

TMR is very effective in improving the capability to control myoelectric prostheses [1–4], but there is little knowledge on the physiology of the newly formed motor units. Such information could provide deeper insights into the neurophysiological events following this surgical procedure and also may prove to be useful for the design of new control algorithms.

Recently, high-density surface EMG systems and sophisticated surface EMG decomposition algorithms made it possible to accurately examine a large number of motor units non-invasively [5]. This allows us to gain insight into characteristics of motor unit populations that influence the control quality of myoelectric prostheses. In this regard the most important characteristic is the distribution of the motor unit action potential (MUAP) on the skin surface, since the TMR procedure aims to facilitate the separation of different movement classes by innervating different parts of the muscle with different nerve branches [2].

The MUAP surface distribution is affected by the distribution of fibres of the unit within the muscle, the composition and thickness of the tissue layer separating the motor unit from the electrode surface, and motor unit size, defined as the number of fibers in the motor unit (also termed innervation number). During the TMR procedure the subcutaneous fat layer is removed, thus the thickness of the tissue layer is reduced. This should result in a surface distribution with smaller area, shorter duration and larger MUAP amplitude [6]. Another possible consequence of TMR is the reduction of motor unit size. The innervation number tends to be greater for muscles that are able to produce more force, and also for muscles not having to perform fine-tuned, precise movements [7]. For this reason, we hypothesize that motor units in the reinnervated trunk muscles in TMR would also be smaller in size, because the corresponding motor neurons originally innervated smaller upper limb muscles.

In this study we aimed to provide a general characterization of motor units in TMR patients with non-invasive electrophysiological methods, and to compare these characteristics with able-bodied controls. To limit the scope of the study, we only considered motor units in the *m*. *pectoralis*, which is easily accessible irrespective of the anatomy of the residual limb.

## Materials and Methods

### Subjects

Five TMR patients and nine able-bodied subjects participated in this study. All patients underwent TMR surgery at the Medical University of Vienna, and had full reinnervation of their targeted muscles at the time of the experiment (Table 1). The detailed patient conditions and the experimental protocol were presented in [8]. All participants read and signed written informed consent form prior to the experiment. The experimental protocol as well as the informed consent form for the TMR patients were approved by the ethics committee “Ethikkommission der Medizinischen Universität Wien” (approval number 1279/2014). None of the able-bodied subjects had any neuromuscular disorders or abnormalities. The experimental protocol and the informed consent form for able-bodied subjects were approved by the “Medizinische Fakultät Ethikkommission der Universitätsmedizin Göttingen” (approval number 9/2/12 and 11/10/14). All experimental protocols were designed and conducted according to the Declaration of Helsinki.

**Table 1:** Age, gender and amputation details of TMR patients.

Subject	Age	Gender	Amputation details	Time since amputation	Time since TMR surgery	TMR site in clavicular head	TMR site in sternocostal part	TMR site in abdominal part
T1	25	Male	shoulder disarticulation, right	3 years and 2 months	10 months	n. musculo-cutaneous	n. medianus	n. medianus
T2	32	Male	Glenohumeral, left	3 years and 2 months	9 months	n. ulnaris	n. medianus	-
T3	40	Male	shoulder disarticulation, left	> 5 years	1 year 5 months	n. musculo-cutaneous	n. medianus	n. medianus
T4	76	Male	Glenohumeral, right	> 5 years	11 months	n. medianus	n. medianus	n. cutaneous antebrachii medialis (sensory)
T5	11	Female	Glenohumeral, left	2 years 3 months	1 year 5 months	n. musculo-cutaneous	n. medianus	n. medianus

Additional information on the TMR patients can be found in [8]. Only TMR sites of the *m*. *pectoralis* are listed. All able bodied subjects were male with the mean age of 30 ± 5.

### EMG acquisition

High-density multichannel surface EMG electrode grids were used for recording. Each grid (ELSCH064NM3, OTBioelettronica, Italy) consisted of 64 electrodes in an 8 by 8 matrix, with an inter-electrode distance of 10 mm. The electrodes were applied on the skin using 1 mm thick two-sided adhesive foam, with holes corresponding to the electrode surfaces. These holes were filled with conductive paste to improve the skin-electrode contact. The donning procedure of the electrode was the same for both TMR patients and able-bodied subjects.

**Fig 1:**
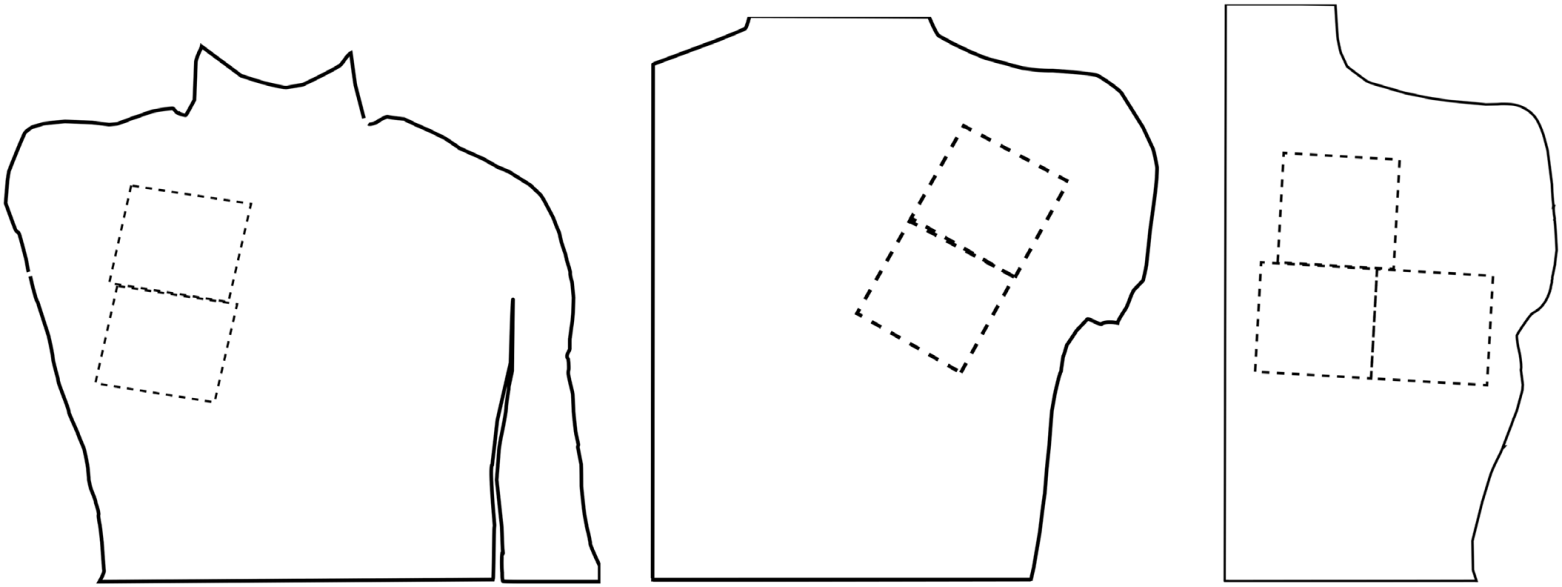
Electrode locations for the TMR patients: T1 and T4 (left), T2 and T5 (middle), T3 (right). The electrode placement of able-bodied subjects was the same as for T2 on the left side and as for T1 on the right side.

The electrode grids were placed on each TMR patient individually, based on reinnervation sites as described by the surgeon (Fig 1). In case of the able-bodied subjects, the electrode placement was equivalent with that of T2. For subject H1 and subjects H5-H9 additional electrodes were placed on the same position of the contralateral side of the body, over the exact muscle area as for patient T1. This was done to investigate whether sidedness and electrode placement have an effect in the statistical analysis, to ensure robustness and statistical validity.

The electrode grids were connected to a 256-channel EMG amplifier (EMGUSB2, OTBioelettronica, Italy). All signals were recorded in monopolar mode, band pass filtered with cut off-frequencies of 3–500 Hz, and sampled at 2048 Hz with a 12 bit A/D converter. The cut-off frequencies for able-bodied subjects were set to 10–900 Hz.

### Experiment protocol

The TMR patients were prompted to perform the following tasks (movement classes) using their phantom limb: hand opening, hand closing, wrist extension, wrist flexion, thumb adduction, thumb abduction, pronation, supination, elbow extension, elbow flexion. The order of the attempts was randomized. Patient T1 was able to sustain the contractions for 10 s with 5 s rest between attempts, the attempts of all other TMR patients lasted 5 s. T1, T4 and T5 performed 8 task attempts twice, T2 performed two task attempts twice and the other three once (5 tasks in total), whereas T3 had two attempts for each of the 10 performed tasks.

The able-bodied subjects performed three types of contractions involving *m pectoralis*: sustained contraction at low force level, sustained contraction at medium force level, and a force ramp up from relaxation to medium force level followed by a ramp down. To provide similar conditions as for the TMR group, force levels were not measured and the subjects received no visual feedback about the force they were exerting. Each attempt lasted 30 seconds, followed by rest for at least 5 seconds. Neither patients nor subjects reported fatigue during or after the experiment.

### Signal processing

#### Decomposition

The recorded EMG signals were decomposed using the Convolution Kernel Compensation (CKC) algorithm [9], an automatic surface EMG decomposition technique. The decomposition did not require pre-filtering to eliminate the ECG artifacts present in the signals, because these are inherently recognized by the algorithm as a separate firing sequence [8]. For T1, T2 and T3 patients the signals recorded from each grid were decomposed separately, since there was no clear spatial association between them. For all other subjects the two grids were jointly decomposed to increase the number of channels and thus decomposition efficiency [5,10].

#### Motor unit discharge statistics

We calculated the mean InterSpike Interval (ISI)–the time interval between two consecutive spiking instants—and the Coefficient of Variation (CoV)–defined as the mean ISI divided by the standard deviation of ISIs—for each decomposed spike train. The ISIs contained outliers due to long pauses in the activity, especially for the TMR patients who were less accurate than the able-bodied subjects in maintaining a stable muscle activity. To remove these outliers, we used the 25th percentile (Q1), the 75th percentile (Q3) and the interquartile range (IQR = Q3-Q1) of the train. The upper limit of valid ISIs was Q3+IQR and the lower limit Q1-IQR. We omitted all ISIs beyond these bounds.

#### Motor unit action potentials and derived properties

**Fig 2:**
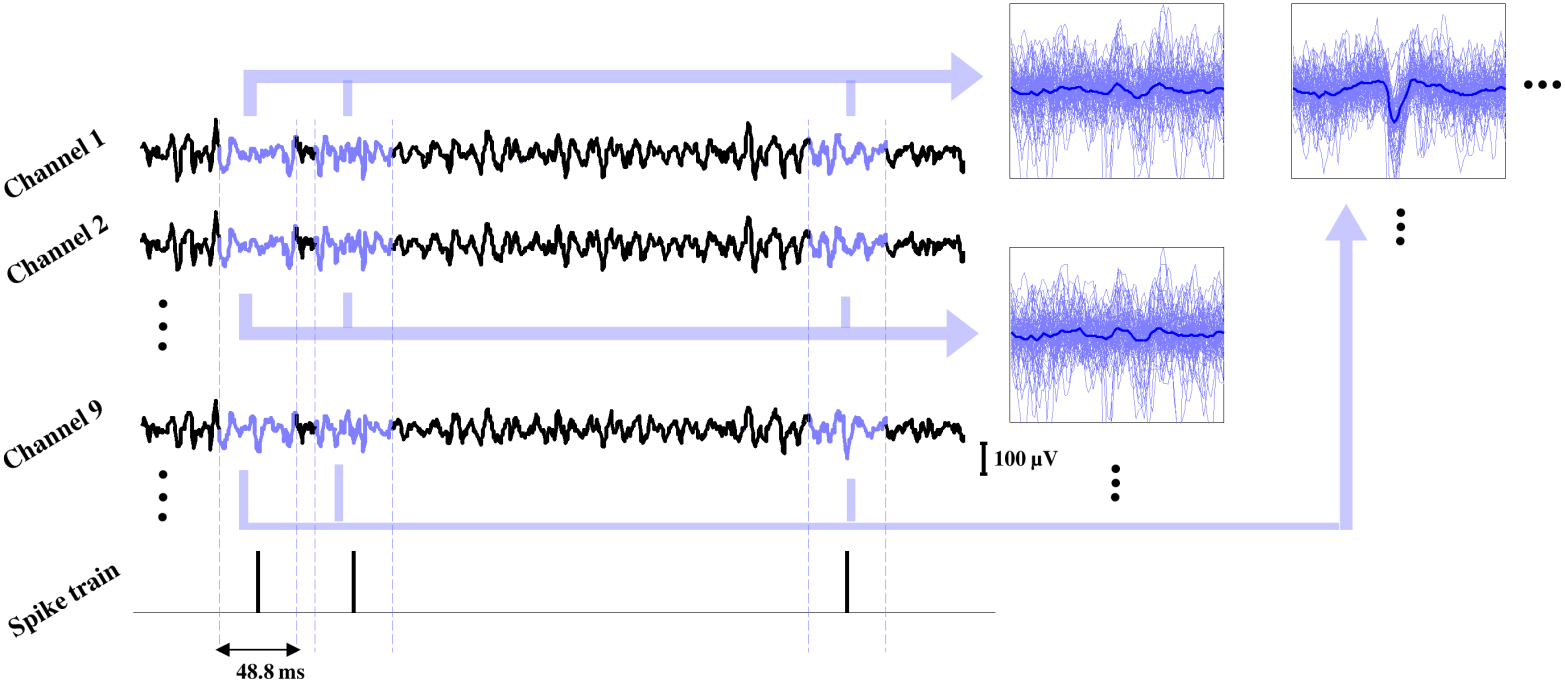
Spike triggered averaging based on the decomposed spike trains. The average waveform around the spiking instants are calculated and organized in an 8 by 8 structure for further processing.

Each decomposed spike train was used to estimate the MUAP waveforms by spike-triggered averaging on each channel (Fig 2). We averaged each channel in a 100 sample (48.83 ms) window, centered on each spiking instant. As a result, each motor unit was characterized by a MUAP over all channels of the recording matrix. To eliminate the influence of ECG artifacts on the spike triggered averages, the EMG signals were high-pass filtered. Although a cut-off frequency of 30 Hz was suggested by [11], for some subjects we observed some ECG related artifacts after filtering, thus a fourth order Butterworth filter with a cut-off frequency of 50 Hz was applied for all subjects.

**Fig 3:**
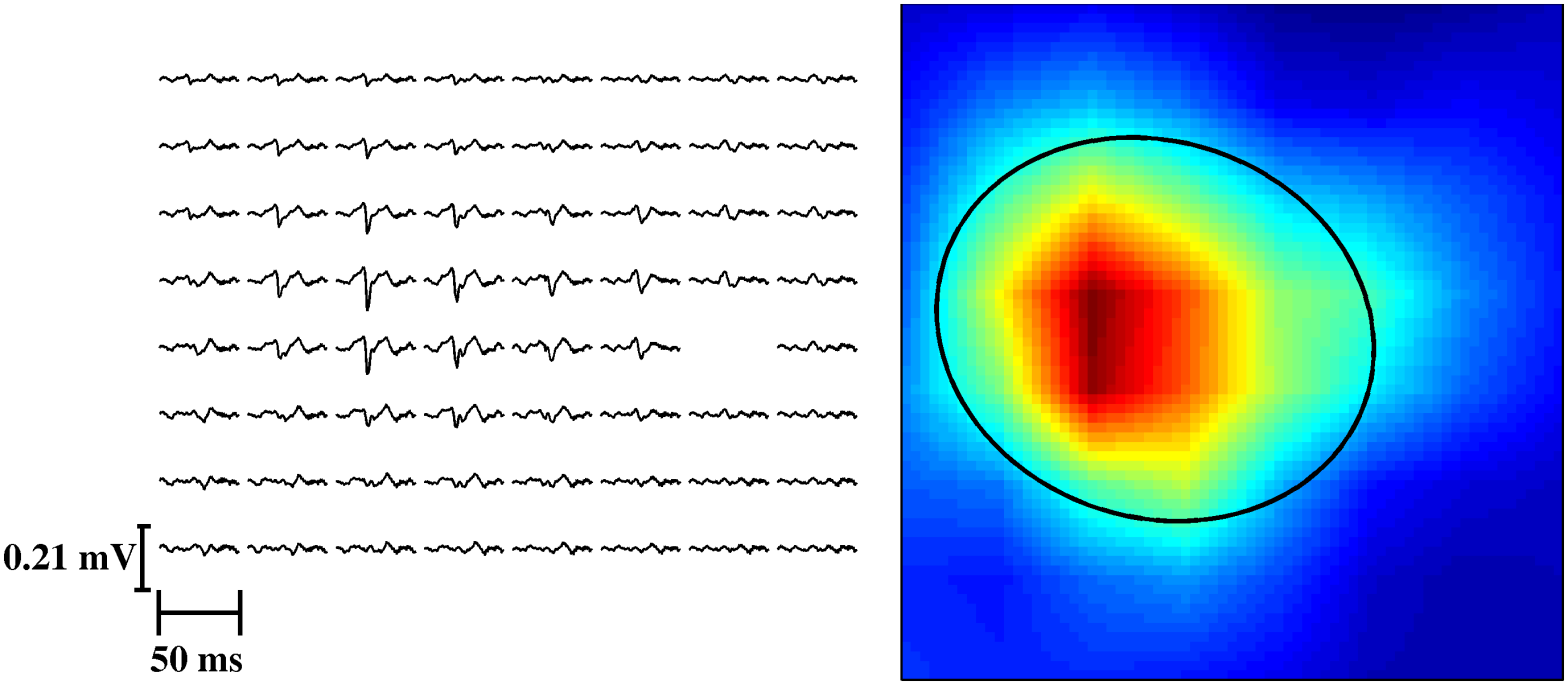
Motor unit action potentials of a decomposed motor unit of subject T3 in all the channels (left) and the corresponding interpolated motor unit RMS map (right). One channel without a MUAP shape was contaminated by signal artefacts, and was excluded from the analysis (blank in the figure). The ellipse fitted on the RMS map of the motor unit is drawn in black on the right. Based on this fitting the motor unit in this example had a normalized MUAP surface area of 0.3.

The territory of a motor unit can be defined as “the subset area of the total muscle cross sectional area that encloses all the fibers belonging to a single motor unit” [10,12,13]. A direct measure of the territory is therefore not possible *in vivo* and indirect approaches are needed. We computed a measure associated to the size of the territory, based on the distribution of the electric potential on the skin surface. Specifically, the area on the skin where the MUAP Root Mean Square (RMS) value was greater than 50% of the maximal MUAP RMS for that motor unit was determined from the RMS spatial mapping (Fig 3). The RMS map was resampled in space to 10 samples per inter-electrode distance using linear interpolation, to gain a sufficient resolution. To each region satisfying the RMS threshold criterion, an ellipse was fit using a least squares fitting algorithm [14]. When the least squares fit corresponded to a conic section other than an ellipse, the region borders were corrected manually and the fitting repeated. The area of the fitted ellipse was normalized by the total area of one grid of electrodes (normalized units) and used as a measure that indirectly indicated the size of the motor unit territory. This measure will be referred to as normalized MUAP surface area.

We also calculated the duration of the action potentials, defined as the time interval that contained more than 80% of the action potential energy, centered on the maximal absolute value. Finally, peak-to-peak amplitudes of the MUAPs were computed.

#### Statistics

For all the statistical comparisons between the TMR and the able-bodied group we used two-way nested ANOVA with an alpha level of 0.05. The random factor “subject” with levels T1-T5, H1-H9 was nested in the fixed factor “group”, containing levels “TMR” and “Healthy”. Because we were only able to record the left side of subjects H2-H4, only the left side of the able-bodied subjects was considered for this comparison.

For comparisons between the two sides within the able bodied group we used the same method, the random factor “subject” with levels H1, H5-H9 was nested in the fixed factor “side”, with levels “left” and “right”.

The descriptive statistics used for reporting were mean and standard deviation.

## Results

**Fig 4:**
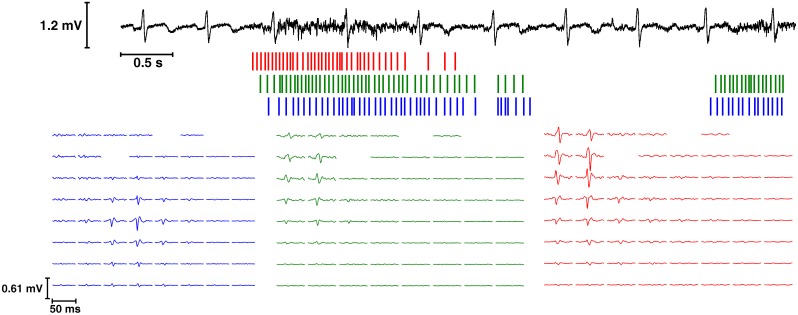
Representation of the decomposition of a single trial for subject T2. Above: one channel of the EMG signal. Below: bar plots of the decomposed spike trains and their MUAPs over the matrix. The colors of the spike trains and the MUAPs are matched. S1 Fig is a black and white version of this figure.

After decomposing each signal with the CKC algorithm, and omitting all the motor units with undetermined surface area, we obtained 270 decomposed spike trains for the TMR patients and 398 for the able-bodied subjects (233 on the left and 165 on the right side, Table 2, Fig 4).

**Table 2:** Number of decomposed spike trains per movement for each subject.

Subject	Side	M1	M2	M3	M4	M5	M6	M7	M8	M9	M10	Mean
**T1**	**Right**	2	5	3	2	6	15	4	7	-	-	5.5
**T2**	**Left**	7	16	7	6	5	6	-	-	-	-	7.8
**T3**	**Left**	9	2	8	4	7	9	5	0	6	10	5.5
**T4**	**Right**	12	8	7	10	8	7	9	5	-	-	8.3
**T5**	**Left**	7	5	7	10	7	5	6	6	-	-	6.6
**H1**	**Left**	19	6	0	-	-	-	-	-	-	-	12.5
**H1**	**Right**	26	4	0	-	-	-	-	-	-	-	15
**H2**	**Left**	3	8	2	-	-	-	-	-	-	-	4.3
**H3**	**Left**	4	5	1	-	-	-	-	-	-	-	3.3
**H4**	**Left**	5	4	7	-	-	-	-	-	-	-	5.3
**H5**	**Left**	11	0	0	-	-	-	-	-	-	-	11
**H5**	**Right**	9	0	0	-	-	-	-	-	-	-	9
**H6**	**Left**	7	5	2	-	-	-	-	-	-	-	4.7
**H6**	**Right**	1	2	2	-	-	-	-	-	-	-	1.7
**H7**	**Left**	24	14	0	-	-	-	-	-	-	-	19
**H7**	**Right**	12	12	0	-	-	-	-	-	-	-	12
**H8**	**Left**	14	15	9	-	-	-	-	-	-	-	12.7
**H8**	**Right**	20	15	5	-	-	-	-	-	-	-	13.3
**H9**	**Left**	28	30	10	-	-	-	-	-	-	-	22.7
**H9**	**Right**	31	21	5	-	-	-	-	-	-	-	19

Each column contains the number of spike trains for a given movement (from Movement 1 (M1) to Movement 10 (M10)). Note that movements were different for each group. For the TMR patients movements were also different for each subject, because not all subjects were able to perform the same tasks with their phantom limb. Thus, each column in this table corresponds to a different movement for each row, and the table only gives a general idea about the number of decomposed spike trains.

**Fig 5:**
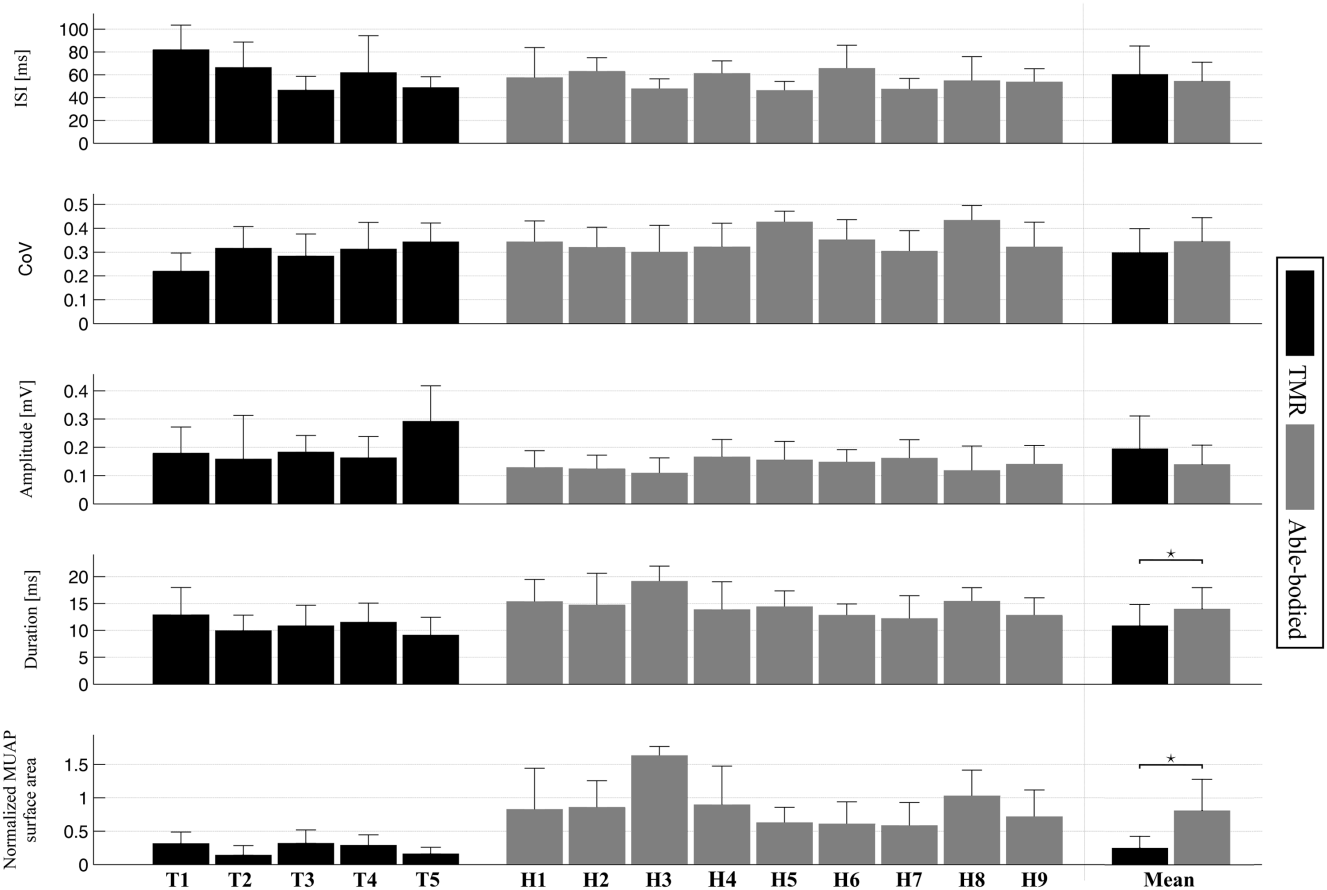
Mean and standard deviation of the investigated motor unit properties of the two groups. TMR patients are shown in black, able-bodied subjects in gray. The group means are depicted on the right side.

The mean ISI values were not significantly different between the two groups (p = 0.41, Fig 5), with average values of 60.47 ± 24.71 ms, for the TMR patients and 54.56 ± 16.30 ms for the able-bodied subjects. The CoV values of the two groups also did not show significant difference (p = 0.12, Fig 5). The means of the two groups were 0.30 ± 0.10 for TMR patients and 0.35 ± 0.10 for able-bodied subjects.

The normalized MUAP surface areas (which are associated to the MU territories) for the TMR group were significantly smaller than for the able-bodied subject group (p < 0.001, Fig 5). The mean normalized area was 0.25 ± 0.17 for TMR patients and 0.81 ± 0.46 for able-bodied subjects. The MUAP durations of the TMR group were significantly smaller than for the able-bodied group (10.92 ± 3.89 ms and 14.03 ± 3.91 ms; p < 0.01, Fig 5). The MUAP peak-to-peak amplitudes were not significantly different between the two groups (0.19 ± 0.11 mV and 0.14 ± 0.06 mV; p = 0.07, Fig 5).

**Fig 6:**
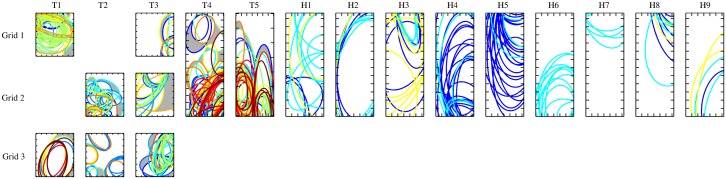
Spatial positions of the motor unit surface areas in each electrode grid (rows) for each subject (columns). Note that for this study, grid 1 for subject T2 and grid 2 for subject T1 were not used, since they were not covering the *m*. *pectoralis*. Surface areas with the same colour in a given grid correspond to motor units identified in the same movement. For able-bodied subjects, one colour represents the same contraction for each subject and for TMR patients the movement classes are different for each individual. The area on the grid occupied by motor units active during only one task is coloured in grey. The tick marks on the image borders denote 1 cm.

We also analyzed the distribution of MUAP surface areas over the surface covered by the electrode matrixes (Fig 6). The motor unit surface areas tended to group in the same regions and therefore overlapped with each other, even if they were active during different contractions. The overall area on the electrode surfaces that contained motor units corresponding to one movement class only was on average 12.08 cm^2^, representing 11.8% of the total electrode surface (Fig 6), with a maximum of 18.9% (17.21 cm^2^) for subject T4.

We did neither observe any statistically significant differences between motor units in the left and right sides of the investigated able-bodied subjects for the motor unit properties above, nor any significant interactions between the factors Side and Subject. The mean values of the two groups were 53.76 ± 16.93 ms and 54.87 ± 14.00 ms for ISI (p = 0.83), 0.35 ± 0.10 and 0.35 ± 0.11 for CoV (p = 0.97), 0.76 ± 0.43 and 0.77 ± 0.41 for normalized MUAP surface area (p = 0.44), 13.72 ± 3.52 ms and 13.30 ± 3.80 ms for MUAP duration (p = 0.75) and 0.14 ± 0.07 mV and 0.13 ± 0.06 mV for MUAP amplitude (p = 0.48) for the left and right side of able-bodied subjects respectively.

## Discussion

In this study we investigated *in vivo* motor units after TMR surgery based on surface EMG decomposition. We examined spiking statistics and motor unit properties of the decomposed spike trains of the *m*. *pectoralis* of five TMR patients and compared them with nine able-bodied controls. We found that MUAP surface area and duration showed a significant difference between the two groups, indicating that motor unit characteristics are influenced by properties of the nerve innervating the new target.

The MUAP surface areas of the respective *pectoralis major* muscles in TMR patients were significantly smaller than those of able-bodied control subjects. Highly localized surface EMG activity is one of the objectives of the TMR procedure, since this facilitates the signal processing required for direct prosthetic control after the procedure. To ensure this spatial separation of EMG activity, different muscle heads of the *pectoralis* are targeted for different branches of the median nerve, sometimes placing subcutaneous fat between the targeted muscle segments [1,2]. We showed that the resulting spatially localized nature of the EMG activity occurs not only on a muscle or muscle-segment level, but also on a motor unit level.

This spatial localization of the motor units in TMR facilitates the detection of areas for direct prosthetic control. However, Fig 6 suggests that motor units active in different tasks have a surface action potential representation that overlaps with units active in other tasks. Therefore, motor units active in different tasks share similar territories in the muscle. This may determine difficulties in optimal electrode placement for maximizing class separation, as illustrated by the fact that the largest area that was activated for a single motion class was at most 18.9% of the total electrode grid surface.

This observation can explain why pattern recognition methods prove to be more effective for TMR patients than proportional control, since direct proportional control may be limited to a small number of spatially separable classes [15]. It also demonstrates why a control approach based on direct neural information obtainable from surface EMG decomposition has even better performance, as found previously [16]. Because spatial separation is not necessary for surface EMG decomposition, even in the case of overlapping motor unit territories, the neural information based control can distinguish between movement classes more accurately.

The reason for the observed difference in the surface EMG representation of the electrical activity of motor units is not necessarily a difference in motor unit territory or size. Our estimate of motor unit territory is also influenced by the composition and depth of the tissue layers between the electrode and the muscle fibers of the motor unit, referred to as the volume conductor effect [6,17]. However, the volume conductor effect should be minimal in the TMR patients because the subcutaneous fat layer over the targeted muscles is eliminated during surgery. Therefore, one of the factors that determine the observed difference in MUAP surface areas between TMR patients and the controls is the thickness of the tissues interposed between the muscle and the recording electrodes. This is also indicated by the longer MUAP durations in the healthy group. Additionally, reinnervation may result in more compact fibre distribution than normal [18], leading to a smaller MUAP surface area. This mechanism is observed, for example, in self-reinnervated muscles, whose cross-sectional size decreases due to an increased fibre density. However, both of the previous factors would lead to an increase in MUAP amplitude in TMR patients, which was not significant in our sample. These differences may reflect an effective smaller size of the motor units after reinnervation. It is highly unlikely that these differences result from differences in electrode placement or sidedness of the TMR patients, since we have found no statistically significant differences between motor units observed in the left and the right side of the able-bodied subjects.

There are indeed several documented changes in motor unit characteristics after reinnervation [19]. These include changes in contractile properties and biochemical changes, all resulting in the new muscle being transformed to fit the original muscle’s properties [20]–this is sometimes referred to as muscle plasticity [21]. Reorganization of the muscle fibers into dense regions was also observed [18], and some findings indicate that this is, present in human TMR patients as well [22]. There is also documented influence of the muscle on the nerve, although typically restrictive or permissive, so that the success of the reinnervation depends on the capability of the muscle to adapt to the new neural input [23]. Based on our results, we speculate that some characteristics of the reinnervated motor units are determined by the motor nerve and therefore represent the physiological innervation of the muscles of the missing limb. It is, however, also possible that the increased spatial localization is a result of the aforementioned reorganization of the muscle units into dense regions, or that both the original motor unit size and the dense reorganization play a role in the resulting spatial localization. The results of this study however do not allow to directly test for these possible mechanisms.

In addition to the motor unit surface areas, we also analyzed the behavior of individual motor units. Although the discharge statistics of the motor units in the two groups were not different, this suggests neither a similarity of motor neuron pools that physiologically innervate different muscles nor adjustments of the motor neuron properties after reinnervation. Because there was no force feedback for any of the groups, we have no information on whether the forces were comparable, and it is possible that different levels of voluntary drive to the motor units resulted in similar discharge characteristics, because the motor neuron pools were different. This limitation, however, is only present for comparing discharge characteristics. Other motor unit characteristics, such as territory, amplitude, etc. do not depend on the neural drive of the motor unit while performing a task, thus these are not affected by the limitations of task comparison between TMR patients and able-bodied subjects.

### Conclusion

The characteristics of reinnervated motor units, as detected by surface EMG decomposition, are different compared to able-bodied controls and present smaller surface areas and shorter action potential durations. The observed distribution of motor unit surface areas in TMR, however, does not necessarily result in highly separable EMG activities among different tasks, as we also observed a large degree of overlap of the surface areas of motor units active in different tasks. This observation explains previous results showing that direct control of multiple degrees of freedom is not always accurate for TMR patients, and more sophisticated control algorithms based on neural information may improve the control performance in some conditions. Our findings indicate that the reason for the observed differences in motor unit characteristics is twofold. On the one hand, muscle fibers of motor units of TMR patients are closer to the recording electrodes due to the reduced fat layer. On the other hand, the reinnervating motor neuron may alter the characteristics of the motor units.

## Supporting Information

S1 Fig Black and white version of Fig 4. (TIF)
