# Supplementary figures and images for: Motor Unit Characteristics after Targeted Muscle Reinnervation
Source: PLoS One. 2016 Feb 22;11(2):e0149772. doi: 10.1371/journal.pone.0149772 (PMC4764766; doi:10.1371/journal.pone.0149772)

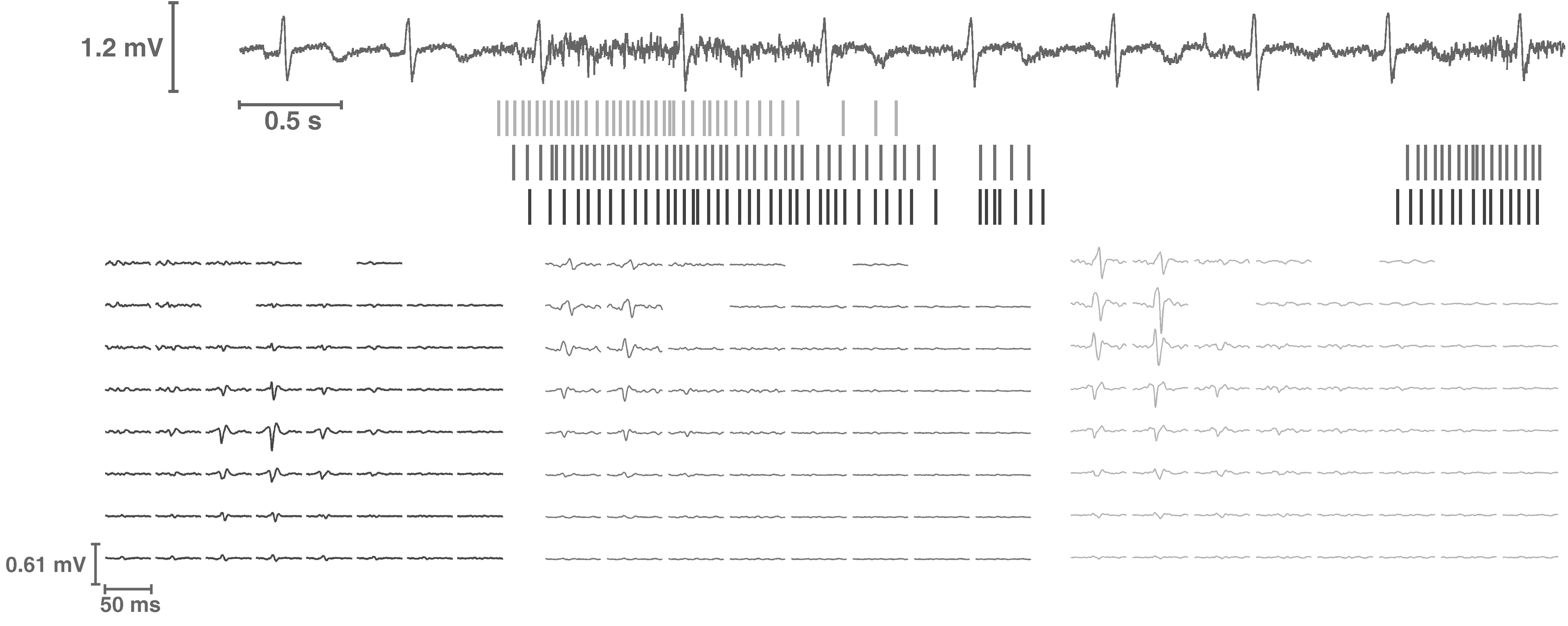

Supplement: S1 Fig — (TIF) [file pone.0149772.s001.tif]
